# A score model based on pancreatic steatosis and fibrosis and pancreatic duct diameter to predict postoperative pancreatic fistula after Pancreatoduodenectomy

**DOI:** 10.1186/s12893-019-0534-4

**Published:** 2019-07-03

**Authors:** Guo Xingjun, Zhu Feng, Yang Meiwen, Jiang Jianxin, He Zheng, Gao Jun, Huang Tao, Zhao Rui, Zhang Leida, Wang Min, Qin Renyi, FACS

**Affiliations:** 1 Department of Biliary-Pancreatic Surgery, Affiliated Tongji Hospital, Tongji Medical College, Huazhong University of Science and Technology, 1095 Jiefang Ave, Wuhan City, Hubei Province 430030 People’s Republic of China; 2 The First Affiliated Hospital of Third Military Medical University, 30 Gaotanyan Str, Chongqing City, 400038 People’s Republic of China; 3 Department of Hepatic-Biliary-Pancreatic Surgery, Renmin Hospital of Wuhan University, Wuhan, China; 4 Department of Hepatic-Biliary-Pancreatic Surgery, Yichang Central People’s Hospital, Yichang, China; 5 Department of Hepatic-Biliary-Pancreatic Surgery, The Affiliated Hospital of Qingdao University, Qingdao, China; 6 Department of Hepatic-Biliary-Pancreatic Surgery, Henan Cancer Hospital, Zhengzhou, China; 7 Department of Hepatic-Biliary-Pancreatic Surgery, The Affiliated Hospital of Guizhou Medical University, Guiyang, China

**Keywords:** Pancreatic fibrosis, Pancreatic steatosis, Postoperative pancreatic fistula, Scoring model

## Abstract

**Purposes:**

To establish a scoring model for the risk of postoperative pancreatic fistula (POPF) following pancreatoduodenectomy (PD).

**Methods:**

PD Patients from 7 institutions in 2 independent sets: developmental (*n* = 457) and validation cohort (*n* = 152) were retrospectively enrolled and analyzed. Pancreatic Fibrosis (PF) and Pancreatic Steatosis (PS) were assessed by pathological examination of the pancreatic stump.

**Results:**

Stepwise univariate and multivariate analysis indicated that pancreatic duct diameter ≤ 3 mm, increased PS and decreased PF were independent risk factors for POPF and Clinically Relevant Postoperative Pancreatic Fistula (CR-POPF). Based on the relative weight and odds ratio of each factor in the POPF, a simplified scoring model was developed. And patients were stratified into high-risk group (22~28 points), medium-risk group (15~21 points) and low-risk group (8~14 points). The receiver operating characteristic curve demonstrated that the Area under the curve for the predictive model was 0.868 and 0.887 in the model design group and the external validation group.

**Conclusions:**

This study establishes a simplified scoring model based on accurately and quantitatively measuring the PS, PF and pancreatic duct diameter. The scoring model accurately predicted the risk of POPF.

**Electronic supplementary material:**

The online version of this article (10.1186/s12893-019-0534-4) contains supplementary material, which is available to authorized users.

## Introduction

Pancreatoduodenectomy (PD) is the optimal treatment for most malignant and benign neoplasms of the pancreatic head and periampullary region. Serious postoperative complications raise concerns for surgeons [1], along with surgical technical difficulty. Postoperative pancreatic fistula (POPF) is one of the most common complications [2]. POPF not only may result in massive intra-abdominal hemorrhaging and severe intraperitoneal infection [3, 4], but is also the most important determinant of death after PD [5]. Surgeons and researchers have attempted to develop various strategies to decrease the incidence of pancreatic leakage after PD such as anastomosis of the pancreas and intestine or placing a tube to support the pancreatic duct [6–8]. Despite many such attempts, the incidence of POPF following PD is still in the 10–28% range [9]. It has been well known that some chronic morbidities, such as alcohol and smoking, contribute to diverse conditions in each patient, especially in regards to the texture of the pancreas [10, 11]. Questions have been raised as to whether pancreatic texture is a potential factor causing high incidence of POPF and if individualized management based on pancreatic texture might change the status quo.

It is believed that the softness or hardness of the pancreas is related to POPF. A soft pancreas makes performing anastomosis of the pancreas and intestine more difficult and makes it easier for the suture line to tear pancreatic tissue. Pancreatic texture can also affect pancreatic exocrine function [12]. The robustness of pancreaticojejunostomy and pancreatic exocrine function both contribute to the occurrence of pancreatic fistula after surgery [13]. However, current research is controversial as to whether a hard pancreas can decrease, or a soft pancreas increase, the incidence of POPF [14–16]. The controversy is not surprising because there are no consensus criteria for quantitatively evaluating pancreatic texture, and when assessed subjectively by each operator, results may differ in perceptions of softness and hardness. Moreover, the cut-off for defining a soft or hard pancreas is also unclear. Using preoperative imaging modalities (CT or MRI) to assess the texture of the pancreas has been attempted. However, imaging modalities only indirectly reflected the texture of the pancreas and can be impacted by many factors. For example, artifacts in MRI can significantly interfere with the evaluation of the fat content of the pancreas.

The softness or hardness of pancreatic texture is determined by the composition of the pancreatic parenchyma which is influenced by the balance of fibrous and fatty tissue [17, 18]. Therefore, pathological analysis of the pancreatic microstructure is the most accurate method to assess pancreatic texture. The aim of this study was to investigate the correlation between the microscopic pathological structure of the pancreas, including level of pancreatic fibrosis (PF) and pancreatic steatosis (PS), and the incidence of POPF after PD in a multicenter retrospective study.

## Methods

### Ethics statement

This study was approved by the Human Research Ethics Committees at the Huazhong University of Science and Technology and was carried out in accordance with the principles embodied in the Declaration of Helsinki. Informed consent for the use of the specimen was obtained from all participants.

### Retrospective study population and patient selection

Seven pancreatic surgery centers contributed to the study (see the institutional affiliations of the authors). The patients’ data from January 2014 to December 2017 were collected and analyzed retrospectively. The eligibility criteria included age greater than 18 years old; died within 1 week. A total of 609 consecutive patients were included in the study for risk factor analysis and the development of a risk score. The risk score was calculated retrospectively after the review of patient records. The patients were represented in the study by code numbers and their personal data were concealed.

### Surgical technique and perioperative management

Each patient was placed in the supine position. The Open Pancreaticoduodenectomy.

(OPD) usually required a long midline incision or straight incision through right rectus abdominis. The Laparoscopic Pancreaticoduodenectomy (LPD) required drilling five small holes in the patient’s abdomen. The supporting stent of the pancreatic duct was placed in some of the patients. Closed rubber drainage tubes were placed near the pancreatic anastomosis and choledochojejunostomy sites. Pancreatic texture and pancreatic duct diameter were assessed by the surgeons.

Broad-spectrum antibiotics (Paclitaxel sulbactam sodium) were intravenously administered to all patients for 3 days after surgery. If there was a clear sign of infection, the period of antibiotic treatment was prolonged. All patients received an H_2_ blocker every 12 h intravenously during the period after surgery when there was no oral intake. After 5d of postoperative octreotide treatment by a 24 h intravenous pump, octreotide dosing was switched to subcutaneous administration (100 mg every 6 h for 7d). The amount of fluid drainage from the peripancreatic site was measured daily and the amylase levels of serum and fluid drainage were measured on postoperative day 1, 3, 5, 7 and 10. On postoperative day 7 an abdominal CT scan was performed and the peripancreatic drainage tube was removed, provided there was no evidence of leakage or fluid collection.

### Hematoxylin and eosin analysis of paraffin embedded sections

The paraffin embedded sections from the pancreatic stump specimens were stained with hematoxylin and eosin (HE) and reviewed by two experienced pathologists who were blinded to the surgical outcome. Based on the criteria of Klopper and Maillet [19], pancreatic fibrosis and pancreatic steatosis was evaluated. The degree of intralobular and interlobular fibrosis was separately scored from 0 to 6 and the total score (0–12) was calculated. According to the total score, fibrosis was classified into normal (Grade 0, score 0–3), mild fibrosis (Grade 1, score 4–6), moderate fibrosis (Grade 2, score 7–9) and severe fibrosis (Grade 3, score 10–12). The degree of pancreatic fat infiltration was assessed based on the percentage of the interlobular fat to total interlobular space and the percentage of the intralobular fat to total intralobular space. The sum of the intralobular and interlobular fat percentages was calculated. Pancreatic steatosis was graded into 4 categories: normal (Grade 0: 0–10%), mild lipomatosis (Grade 1: 11–40%), moderate lipomatosis (Grade 2: 41–70%), and severe lipomatosis (Grade 3: 71–100%).

### Definition of pancreatic fistula

The International Study Group of Pancreatic Fistula (ISGPF) definition on POPF was used in our study [20].

### Statistical analysis

Statistical analyses were performed using SAS version 9.4 (SAS Inc., Chicago, IL, USA). Univariable analysis was performed on the various parameters of the POPF and non-POPF, CR-POPF and non-CR-POPF groups. The variables were then selected into multivariable logistic regression models, with forward stepwise selection procedures. Statistically significant differences were defined as *p* < 0.05*.*

## Results

### Demographic and Clinicopathologic characteristics of cohort

Patient demographics and perioperative characteristics are listed in Additional file 1: Table S1. During the study period, a total of 609 patient underwent PD at the 7 participating centers. The patients were comprised of 383 men (62.9%) and 226 women (37.1%), with a median age of 54.6 years (range: 18–82 years). The median BMI was 22.0 kg/m^2^ (range: 15.1–34.0 kg/m^2^). Most patients had ASA scores of II (74.1%) or III (17.2%). The mean preoperative TBIL was 138.2 μmol/L (range: 3.7–570.2 μmol/L) and the mean preoperative DBIL was 72.3 μmol/L (range: 0.5–278.4 μmol/L). The mean preoperative ALT was 147.0 U/L (range: 1.2–1099.0 U/L); the mean preoperative AST was 113.2 U/L (range: 9.0–1628.0 U/L). The mean preoperative CT Hu value was 39.6 (range: 16.3~65.7). Prior abdominal surgery had been performed in 23.6% of the patients. Preoperative jaundice and abdominal pain were the most common clinical symptoms. The preoperative diagnoses of mass location were pancreatic head (66.3%), duodenum (7.2%), biliary duct (17.6%), and ampulla (9.0%).

Intraoperative details are shown in Additional file 1: Table S1. Laparoscopic pancreaticoduodenectomy was performed in 79 patients (13.0%) and OPD in 530 patients (87.0%). The median operative time was 398.8 min (SD = 120.1 min). The methods of pancreatic remnant anastomosis included PJ (85.6%) and PG (14.4%). Stenting of the pancreatic duct was required in 554 cases (92.0%). The median estimated intraoperative blood loss was 489.6 mL (SD = 345.7 mL). Intraoperative blood transfusions were needed in 129 patients (21.2%); the median estimated volume of intraoperative blood transfusion was 173.0 mL (SD = 231.5 mL).

Postoperative details are shown in Additional file 1: Table S1. According to the ISGPF, 73 patients (12.0%) had a grade A POPF; these patients were treated conservatively and fed orally without additional intervention. Grade B POPF occurred in 41 patients (6.7%) and grade C POPF in 27 patients (4.4%).

Pathological outcomes are shown in Additional file 1: Table S1. Lesions were caused by chronic inflammation in 82 cases (13.5%), cystic neoplasia of the pancreas in 82 cases (13.5%), ampullary carcinoma in 55 cases (9.0%), duodenal lesions in 43 cases (7.1%), cholangiocarcinoma in 107 cases (17.6%), and pancreatic carcinoma in 245 cases (40.2%). The grade of pancreatic fibrosis of the pancreatic stump was normal in 168 cases (27.6%), mild in 181 cases (29.7%), moderate in 204 cases (33.5%), and severe in 56 cases (9.2%). The grade of pancreatic steatosis of the pancreatic stump was normal in 292 cases (47.9%), mild in 205 cases (33.7%), moderate in 95 cases (15.6%), and severe in 17 cases (2.8%).

### Univariate analysis and correlation of variables with POPF

A total of 640 patient was identified, with 31 being excluded from analysis because of missing data. Postoperative pancreatic fistula occurred in 141 of the 609 patients (23.2%). Univariate analyses of the variables and their association with POPF are shown in Table 1. In total, the 10 variables (BMI, AST, CT Hu value, pancreatic texture, pancreatic duct diameter, surgery approach, operating time, histopathology, PF and PS) were significantly different between the no-POPF group and POPF group. Univariate analyses of the variables and their association with CR-POPF are shown in Table 3. In total, the 4 variables (pancreatic texture, pancreatic duct diameter, PF and PS) were significantly different between the no-CR-POPF group and CR-POPF group.

**Table 1:** Univariate Analysis and Relationship of Variables With POPF

Variables	No	POPF		*P*	CR-POPF		*P*
		no	yes		no	yes	
Sex: Male / Female	383/226	285/183	98/43	0.064	340/201	43/25	0.173
Age: ≤55/> 55	300/309	227/241	73/68	0.281	265/276	35/33	0.699
BMI: ≤23/> 23	327/282	268/200	59/82	0.001	290/251	37/31	0.900
Diabetes: No/Yes	551/58	423/45	128/13	0.889	489/52	62/6	0.546
Abdominal surgery history: No/Yes	465/144	352/166	133/28	0.227	403/128	52/16	0.917
RBC: normal / abnormal	392/217	305/163	87/54	0.451	348/193	44/24	0.951
PCT: ≤0.5/> 0.5 ng/mL	374/235	288/180	86/55	0.907	231/210	43/25	0.095
ALT: ≤40/> 40 U/L	204/405	166/302	38/103	0.060	181/360	23/45	0.952
AST: ≤40 / > 40 U/L	229/380	189/279	40/101	0.009	203/338	26/42	0.909
Platelet: normal / abnormal	466/143	362/106	104/37	0.378	408/127	52/16	0.969
Total Bilirubin: ≤171 / > 171 μmol/L	424/185	334/134	90/51	0.088	377/164	47/21	0.923
Direct Bilirubin: ≤110 / > 110 μmol/L	350/259	272/196	78/63	0.555	311/230	39/29	0.913
CT Hu value: ≤40Hu / >40Hu	423/186	336/132	87/54	0.023	371/110	52/16	0.903
Surgery approach: OPD / LPD	530/79	397/71	133/8	0.003	467/74	63/5	0.143
Pancreatic texture (evaluation by surgeon)							
soft	318	211	107	< 0.001	276	42	< 0.001
middle	205	174	31		179	26	
hard	86	83	3		86	0	
Operating time: ≤240 min / > 240 min	273/336	222/246	51/90	0.018	241/300	32/36	0.695
Pancreatic duct diameter: ≤3 / > 3 mm	312/297	206/262	106/35	< 0.001	298/243	14/54	< 0.001
Intraoperative bleeding: ≤400 / > 400 mL	387/222	298/170	73/50	0.905	244/197	43/25	0.221
Intraoperative blood transfusion: ≤400 / > 400 mL	563/46	432/36	131/10	0.813	500/41	63/5	0.859
Histopathology							
Chronic inflammation	77	63	14	< 0.001	68	9	0.074
Cystic neoplasia of the pancreas	82	63	19		73	9	
Ampullary carcinoma	55	42	13		49	6	
Duodenal lesions	43	22	21		38	5	
Cholangiocarcinoma	107	68	39		95	12	
Pancreatic cancinoma	245	210	35		218	27	
Pancreatic Fibrosis							
0	168	79	89	< 0.001	126	42	< 0.001
1	181	152	29		158	23	
2	204	182	22		201	3	
3	56	55	1		56	0	
Pancreatic Steatosis							
0	292	258	34	< 0.001	288	4	< 0.001
1	205	156	49		181	24	
2	95	48	47		64	31	
3	17	6	11		8	9	

Data are expressed as whole numbers, with *P* values from Fisher exact test

### Multivariate analysis and correlation of variables with POPF

Next, the 609 patients were divided into two groups. Data from 3/4 of the patients (*n* = 457) was used for a multivariate analysis and design model and the remaining 1/4 patient’s (*n* = 152) data was used for external validation for the logistic regression model. In the multivariate logistic regression analysis between the no-POPF group and POPF group, pancreatic duct diameter, PS and PF were independent factors with significance (Table 2). Patients with a pancreatic duct diameter > 3 mm were 0.341-fold risk of POPF compared with a diameter ≤ 3 mm. Both PS and PF are ordinal variables with numerical values from 0 to 3. The risk of POPF for each additional grade of PS is 1.621 times the lower grade. The risk of POPF for each additional grade of PF is 0.709 times the lower grade. In the multivariate logistic regression analysis between the no-CR-POPF group and CR-POPF group, pancreatic duct diameter, PS and PF were independent factors with significance (Table 2).

**Table 2:** Multivariate logistic regression models of independent risk factors for POPF

	Odds ratio	95% CI	*P* value
No POPF VS. POPF			
Pancreatic duct diameter	0.34	0.19–0.59	< 0.001
Pancreatic Steatosis	1.62	1.76–3.31	< 0.001
Pancreatic Fibrosis	0.71	0.52–0.96	0.03
No CR-POPF VS. CR-POPF			
Pancreatic duct diameter	3.33	1.96–5.64	< 0.001
Pancreatic Steatosis	0.78	0.67–0.91	0.01
Pancreatic Fibrosis	2.42	1.47–3.97	< 0.001

### POPF risk score model

**Fig. 1:**
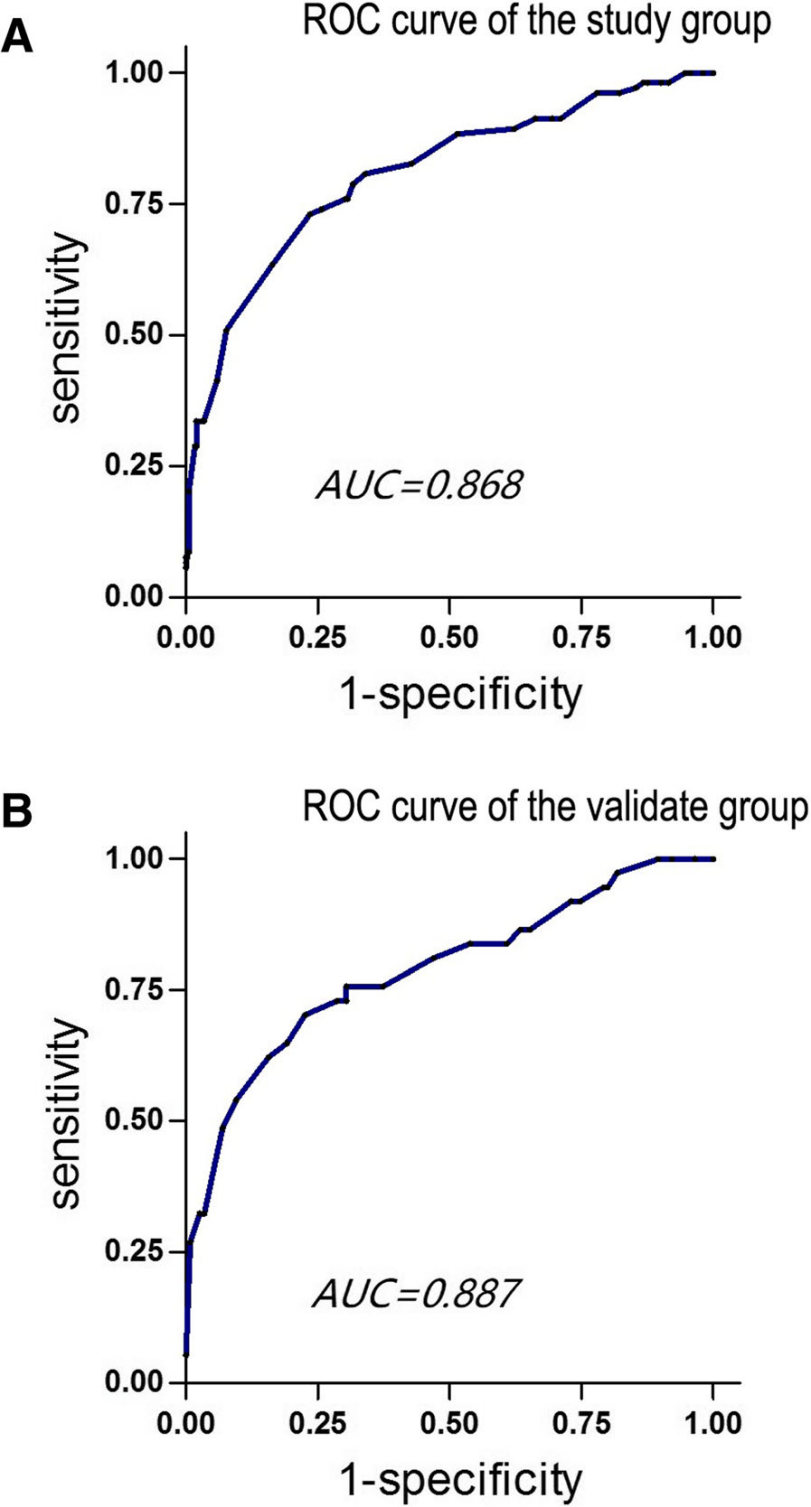
Receiver operating characteristic curve. **a**: Receiver operating characteristic (ROC) curve for the predictive scoring model of the study group. Area under the receiver operator characteristics curve was 0.868. **b**: ROC curve for the predictive scoring model of the validate group. Area under the receiver operator characteristics curve was 0.88

The weighting of each risk factor can be calculated based on the coefficient of each variable in the multivariate linear regression analysis. An odds ratio was also revealed in the multivariate linear regression analysis (Table 2). The authors assumed each risk factor contributes to POPF, with respect to weighting and odds ratios, and each variable was given a certain value by calculating the product of weighting times odds ratio (Table 3). Therefore, a simplified scoring model was generated, and 3 variables, pancreatic duct diameter, PS and PF, were included. The scores ranged from 8 to 28 and were stratified into into high-risk group (22~28 points), medium-risk group (15~21 points) and low-risk group (8~14 points) (Table 3). The receiver operating characteristic (ROC) curve (Fig. 1a) in the model design group shows that the Area under the curve (AUC) of the score was 0.868. The ROC curve in the external validation database (Fig. 1b) shows that the AUC of the score was 0.887.

**Table 3:** Risk scoring model for POPF

Risk Factor	Risk Factor Weight	OR	Points contributed
Pancreatic duct diameter			
> 3 mm	2.81	1.00	2
≤ 3 mm	2.81	2.93	8
Pancreatic Steatosis			
0	3.79	1	4
1	3.79	1.62	6
2	3.79	2.62	10
3	3.79	4.25	16
Pancreatic Fibrosis			
3	1.60	1	2
2	1.60	1.41	2
1	1.60	1.99	3
0	1.60	2.81	4

Score max = 28, score min = 8

Low-risk: 8~14

Medium-risk: 15~21

High-risk: 22~28

## Discussion

Postoperative pancreatic fistula continues to be a challenge in postoperative management. It increases patient distress, prolongs hospitalization and escalates the medical cost. In high volume institutions, POPF rates are high and are a primary detrimental factor causing modality and mortality. In this study, the overall incidence of POPF after PD was 23.2%, which was similar to the POPF rate in several large multicenter cohort studies [21–23].

Incorporating various risk factors into a scores model that predicts POPF risk has been reported by various groups [21, 24–31]. However, most of these models are built with single-center data, either not having been externally validated or having poor to fair performance at external validation, which restricts the applicability for clinical practice. The Fistula Risk Score (FRS) created by Callery and colleagues [27], which is based on gland texture, pancreatic duct diameter, intraoperative blood loss and definitive pathology, is the most cited and widely accepted POPF prediction model. It has been reported that the internal validation of AUC for Models I, II, and III are 0.936, 0.938 and 0.942, respectively. However, Olga and colleagues’ [32] recent research, based on NSQIP data, showed a Modified FRS (without blood loss) prediction model with poor external validation performance, where the AUC was only 0.62. In addition, included the most recently published Alternative FRS (without blood loss) study [33], these models rely heavily on pancreas texture to stratify risk groups. Despite pancreas texture being widely recognized as a strong prediction factor for POPF and applied universally in clinical practice, the subjective assessment is affected by many factors including but not limited to perceptions and experience, ultimately limiting its predictive value. To address these limitations, a new predictive model that can objectively reflect pancreas consistency and incorporate other risk factors, as well as predict and stratify POPF risk, needs to be developed. In this study, pancreatic texture was accurately evaluated based on the microstructure of the pancreas. In addition, the scoring model contain 3 main factors. We give each factor the appropriate score based on the relative weight of the three factors, which is more reasonable than the average score given in previous studies.

Pancreatic fibrosis and PS are well known for affecting the consistency of the pancreas. In this study’s systematic reviews, research indicated that low PF and high PS were risk factors for pancreatic leakage after pancreatic resection. One of the hypotheses is that the fiber and adipose tissue replace the normal pancreatic parenchyma destroying the normal microstructure of the pancreas, ultimately leading to changes in pancreatic texture [34–36]. Fatty infiltration in the pancreas makes the tissue more fragile, making anastomosis of the pancreas and intestine more easily disrupted, while deposition of fibrous tissue in the pancreas makes the tissue firm and reduces the complications in surgical operation [37]. Another hypothesis is that a fibrotic pancreas can decrease the incidence of POPF because of decreased pancreatic exocrine function. This study revealed that patients with severe PF had lower incidences of pancreatic fistula following PD and those with severe pancreatic fat infiltration had higher incidences. These results are consistent with several reported studies [16, 38, 39]. Many studies also concluded pancreatic duct diameter to be a major determinant of POPF [26, 40–42]. In this study, the data revealed that a pancreatic duct diameter less than 3 mm was a risk factor, with an odds ratio of 2.93 in POPF after PD. It is proposed that smaller pancreatic ducts are more prone to occlude or dehisce after a challenging duct-to-mucosa anastomosis procedure and obstructed or leaking pancreatic juice might corrode the pancreatic intestinal anastomosis, leading to pancreatic leakage. Wada et al. [43] suggested that using surgical microscope magnification for better visualization would decrease POPF rates.

The authors of this study were able to successfully develop and validate a POPF prediction model. This simplified scoring model consists of only three variables: PS, PF and pancreatic duct diameter. Using only three variables was shown to not sacrifice predictive capacity, where the AUC of the ROC curve for the model design group and external validation group were 0.868 and 0.887, respectively. This predictive model stratifies patients into three risk groups.

The limit of this study is that the three key variables used in the predictive model are intraoperative variables, limiting the model’s applicability for preoperative patient counseling. Next we should carry on a multicenter, prospective study to analyze whether this scoring system can guide clinicians in choosing the right pancreatic anastomosis. There are many ways to reconstruction the pancreas, but there is no uniform opinion on the choice of anastomosis. And we should also evaluate whether or not we can get accurate data of pancreatic fibrosis, pancreatic steatosis and pancreatic duct diameter during the surgery. After establishing the scoring system, we need to evaluate whether the surgeon can choose a more reasonable match according to the score.

## Conclusion

Changes in pancreatic microstructure, such as severe fatty tissue infiltration in the pancreas, decreased pancreatic fibrosis and a smaller pancreatic duct diameter are the major risk factors for POPF following PD. A new predictive model can stratify patients into different POPF risk groups.

## Additional files

Additional file 1: **Table S1.** Demographic and Clinicopathologic Characteristics of Cohort (DOC 70 kb)

## Data Availability

The datasets used and analysed during the current study are available from the corresponding author on reasonable request.

## Abbreviations

ALT: Alanine aminotransferase
AST: Aspartate aminotransferase
BMI: Body Mass Index
CP: Chronic Pancreatitis
CR-POPF: Clinically Relevant Postoperative Pancreatic Fistula
DP: Distal Pancreatectomy
HE: hematoxylin and eosin
LPD: Laparoscopic Pancreaticoduodenectomy
OPD: Open Pancreaticoduodenectomy
PC: Pancreatic Carcinoma
PCT: Procalcitonin
PD: Pancreatoduodenectomy
PF: Pancreatic Fibrosis
PG: Pancreaticogastrostomy
PJ: Pancreaticojejunostomy
POPF: Postoperative Pancreatic Fistula
PS: Pancreatic Steatosis
RBC: Red blood cell
