# Supplementary material for: A score model based on pancreatic steatosis and fibrosis and pancreatic duct diameter to predict postoperative pancreatic fistula after Pancreatoduodenectomy
Source: BMC Surg. 2019 Jul 3;19:75. doi: 10.1186/s12893-019-0534-4 (PMC6610813; doi:10.1186/s12893-019-0534-4)
Supplement: Supplementary file 1 — Table S1. Demographic and Clinicopathologic Characteristics of Cohort (DOC 70 kb) [file 12893_2019_534_MOESM1_ESM.doc]

**Supplemental Table 1. Demographic and Clinicopathologic Characteristics of Cohort**

| **Demographics** | **Num. (Percent, %)** | **Mean（SD）** | **Min~Max** |
| --- | --- | --- | --- |
| **Sex (M/F)** | 383 (62.9%) / 226 (37.1%) |  |  |
| **Age (years)** |  | 54.6 (11.1) | 18.0~82.0 |
| **BMI** |  | 22.0 (3.2) | 15.1~34.0 |
| **Diabetes** | 58 (9.5%) |  |  |
| **Abdominal surgery history** | 144 (23.6%) |  |  |
| **RBC** |  | 4.0 (0.6) | 2.2~5.8 |
| **PCT** |  | 0.4 (1.2) | 0.1~4.2 |
| **ALT** |  | 147.0 (162.8) | 1.2~1099.0 |
| **AST** |  | 113.2 (152.3) | 9.0~1628.0 |
| **Platelet** |  | 238.2 (89.2) | 86.0~545.0 |
| **TBIL(μmol/L)** |  | 138.2 (135.2) | 3.7~570.2 |
| **DBIL(μmol/L)** |  | 72.3 (135.2) | 0.5~278.4 |
| **CT Hu value** |  | 39.6 (7.9) | 16.3~65.7 |
| **Preoperative diagnosis** |  |  |  |
| Pancreatic head mass | 404 (66.3%) |  |  |
| Duodenum mass | 43 (7.2%) |  |  |
| Biliary duct mass | 107 (17.6%) |  |  |
| Ampullary mass | 55 (9.0%) |  |  |
| **ASA score, N (%)** |  |  |  |
| I | 51 (8.4%) |  |  |
| II | 451 (74.1%) |  |  |
| III | 105 (17.2%) |  |  |
| IV | 2 (0.3%) |  |  |
| **Surgery approach** |  |  |  |
| OPD | 530 (87.0%) |  |  |
| LPD | 79 (13.0%) |  |  |
| **Pancreatic texture** |  |  |  |
| soft | 318 (52.2%) |  |  |
| middle | 205 (33.7%) |  |  |
| hard | 86 (14.1%) |  |  |
| **Operating time** |  | 398.8 (120.1) | 178.0~956.0 |
| **Intraoperative bleeding** |  | 489.6 (345.7) | 50.0~2500.0 |
| **Intraoperative blood transfusion** |  | 173.0 (231.5) | 0.0~1800.0 |
| **Pancreatic anastomosis** |  |  |  |
| PG / PJ | 88 (14.4%) / 521(85.6%) |  |  |
| end-to-end / end-to-side | 141 (23.2%) / 468 (76.8%) |  |  |
| Suturing / no-suturing | 457 (75.0%) / 152 (25.0%) |  |  |
| **Stent of pancreatic duct** | 554(92.0%) |  |  |
| **Histopathology** |  |  |  |
| Chronic inflammation | 77 (12.6%) |  |  |
| Cystic neoplasia of the pancreas | 82 (13.5%) |  |  |
| Ampullary carcinoma | 55 (9.0%) |  |  |
| Duodenal lesions | 43 (7.1%) |  |  |
| Cholangiocarcinoma | 107 (17.6%) |  |  |
| Pancreatic cancinoma | 245 (40.2%) |  |  |
| **Pancreatic Fibrosis** |  |  |  |
| Normal (Grade 0: score 0-3) | 168 (27.6%) |  |  |
| Mild (Grade 1: score 4-6) | 181 (29.7%) |  |  |
| Moderate (Grade 2: score 7-9) | 204 (33.5%) |  |  |
| Severe (Grade 3: score 10-12) | 56 (9.2%) |  |  |
| **Pancreatic Steatosis** |  |  |  |
| Normal (Grade 0: 0-10%) | 292 (47.9%) |  |  |
| Mild (Grade 1: 11-40%) | 205 (33.7%) |  |  |
| Moderate (Grade 2: 41-70%) | 95 (15.6%) |  |  |
| Severe (Grade 3: 71-100%) | 17 (2.8%) |  |  |
| **POPF** |  |  |  |
| A | 73 (12.0%) |  |  |
| B | 41 (6.7%) |  |  |
| C | 27 (4.4%) |  |  |
